# Frailty Index as a Predictor of Operative Safety and Efficacy in Patients Undergoing Laparoscopic Sleeve Gastrectomy

**DOI:** 10.1007/s11695-025-07713-y

**Published:** 2025-03-18

**Authors:** Eliahu Yonathan Bekhor, Boris Kirshtein, Noam Peleg, Nayyera Tibi, Hila Shmilovich, Lisa Cooper, Alex Tatarov, Nidal Issa

**Affiliations:** 1 Rabin Medical Center, Petah Tikva, Israel; 2 Tel Aviv University, Tel Aviv, Israel

## Abstract

**Background:**

Bariatric surgery is an effective treatment for obesity and its associated comorbidities. However, the safety and efficacy of laparoscopic sleeve gastrectomy (LSG) for elderly and frail populations remain uncertain.

**Objectives:**

To validate the efficacy and safety of LSG for elderly and frail patients and to assess its impact on overweight and obesity-related comorbidities.

**Methods:**

A retrospective cohort study of patients undergoing LSG at a university-affiliated single center between 2009 and 2022 from a prospectively maintained database. Patients were categorized into two cohorts based on age and frailty index: elderly vs younger patients and frail vs non-frail groups. Preoperative, perioperative, and postoperative data were analyzed.

**Results:**

Frailty was associated with statistically significantly higher perioperative complications (%, Clavien-Dindo of III/IV, 8 vs 3) and lower treatment success rates (% Excess Body Weight Loss, six-month, year, and two-year, 58 vs 64, 73 vs 82, 72 vs 81, and 63 vs 76, respectively). Age was not shown to alter the safety or efficacy of the operation.

**Conclusion:**

While LSG is a viable option for elderly and frail patients, frailty is a significant predictor of treatment outcomes. A comprehensive assessment of individual factors, including frailty status, is essential for informed decision-making before surgery.

## Introduction

The population is getting older. In 2000, 600 million persons were aged 60 years or older; it is estimated to grow to 2 billion by 2050 [1]. Concurrently, obesity prevalence has increased dramatically and now affects almost one-third of the adult population in North America [2].

In recent years, as knowledge of geriatric patients has accumulated, the focus has shifted from regarding age as a definitive and singular parameter to a wider biological assessment of the patients. This considers their physiological status, as described by various frailty indexes [3–5]. A Systemic review published in 2016 [6] concludes that frailty in the elderly leads to higher rates of perioperative complications in the older population.

In the last four decades, followed by groundbreaking publications [7, 8], bariatric surgery has been widely accepted as the most sustained and effective means to tackle morbid obesity and its co-morbidities [9–11]. Furthermore, bariatric surgeries are proven safe with low 30-day complication rates and highly rare 30-day mortality events [12]. Currently, laparoscopic sleeve gastrectomy (LSG) is the most common bariatric and metabolic surgery procedure, accounting for up to 60% of all bariatric procedures both globally and in the US [13–15].

Although obesity prevalence in elderly patients is similar to its prevalence in the younger population [16], the safety and efficacy of bariatric surgery in this population remain controversial [17–20]. Athanasiadis et al. [21] compared the outcomes between more than a thousand patients over and under 60 years old who underwent bariatric surgery. In concordance with the other cited publications, they showed that older patients do not suffer from high complication rates but generally lose less weight compared to the younger population.

Two main gaps that were identified in the current literature are (i) the correlation between weight loss and obesity-related co-morbidity remission in the elderly population and (ii) the impact of frailty on the procedure’s safety and efficacy.

Considering this, we aimed to assess whether weight loss and age are sufficient predictors of LSG efficacy and safety, or rather, the utilization of the frailty index would prove more accurate.

## Material and Methods

Data on all patients who underwent LSG between 2009 and 2022 in HaSharon Hospital of Rabin Medical Center, a Tel Aviv university-affiliated single center, was included in a prospectively maintained database under IRB approval. All identifying data was coded and saved on a secured hospital computer.

A dedicated bariatric surgeon’s team operated on all patients with similar operative techniques and instruments. The patients were routinely discharged on postoperative day 2 unless any concern arose. A drain was not routinely applied. The patients were followed in an outpatient clinic every 3 months for the first year and yearly thereafter. A dedicated team of dietitians and social workers was routinely involved in the follow-up.

Clinical data, including patient demographics, weight, BMI, co-morbidities, and intraoperative and postoperative courses, was collected from hospitals' databases.

The efficacy of the surgery was evaluated using two parameters: weight loss and improvement or resolution of obesity-related co-morbidities. Improvement in co-morbidities was defined as less medication being needed and/or better equilibrium being achieved [22]. Resolved co-morbidities were defined as no need for medication to treat a certain pre-existing disease.

Pre-operative data, peri-operative course, and long-term outcomes were compared between the Elderly Group (EG), which included patients aged 60 and older, and the Younger Group(YG), which included patients younger than 60. In addition, the Memorial Sloan Kettering Frailty Index (MSK—fi) [3], appendix I, was used to evaluate patients’ frailty.

MSK-FI has emerged as a valuable tool for assessing frailty. The MSK-fi incorporates various health domains, including functional status, nutritional deficiencies, and comorbid conditions, enabling a nuanced assessment of a patient's operative risk. Frailty is strongly associated with postoperative complications, prolonged recovery, and reduced long-term survival [3].

The cutoff for frailty was three or above (Frail group) as per the MSK groups’ study to separate the cohort into frail and non-frail groups.

Statistical analysis was conducted using SPSS® software, version 22. Categorical variables, such as gender distribution and the occurrence of postoperative complications, were analyzed using Chi-squared tests. The results were reported as totals and percentages to facilitate direct comparisons between groups. For continuous variables, including weight, BMI, and excess body weight loss (%EBWL), comparisons were performed using Student’s t-tests. To ensure the validity of these tests, the homogeneity of variances was assessed using Levene’s test. A p-value of less than 0.05 was considered statistically significant. Missing data were excluded from analyses on a per-variable basis. Sensitivity analyses were not explicitly conducted.

## Results

### Elderly Group vs Younger Group

Between 2009 and 2022, 895 patients underwent LSG at our department during the study period. Of these, 85 were older, and 810 were younger patients. The median age was 64 years (IQR, 61 – 66) for the EG and 38 years (IQR, 31—49) for the YG. The median follow-up was 30 months (IQR,16–35).

### Elderly Group vs Younger Group – Demographics – Table 1

**Table 1:** Elderly vs Younger groups: Preoperative demographics and evaluation

Variable	Elderly Group *n* = 85	younger Group *n* = 810	*p*—value
Gender, Female, n (%)	51 (60)	581 (72)	0.02
MSK-FI, mean (SD)	1.68 (1.0)	0.77 (0.95)	< 0.01
Pre-operative Weight, Kg, mean (SD)	119 (20)	120 (20)	0.81
Weight change before operation, Kg, mean (SD)	−1.6 (3.5)	−0.7 (3.7)	0.04
Weight at operation, Kg, mean (SD)	118 (20)	119 (20)	0.58
BMI at operation, mean (SD)	44 (6)	43 (6)	0.57
EBW, Kg, mean (SD)	50 (17)	50 (16)	0.40
Preoperative Diabetes Mellitus, n (%)	42 (49)	197 (24)	< 0.01
Preoperative Hypertension, n (%)	62 (73)	206 (25)	< 0.01
Preoperative Dyslipidemia, n (%)	56 (66)	267 (33)	< 0.01
Preoperative Obstructive Sleep Apnea, n (%)	18 (21)	85 (10)	< 0.01
HbA1c before operation, mean (SD)	6.8 (1.1)	6.5 (1.4)	0.08

*SD* – Standard Deviation, *MSK-FI* – Memorial Sloan Kettering Frailty Index, *KG* – kilogram *BMI* – Body Mass Index, *EBW* – Excess Body Weight, HbA1c – Hemoglobin A1c

Patients in the EG were predominantly females (%, 72 vs 60, *p* = 0.02), had similar operative weight (kg, mean, 118 vs 119, *p* = 0.58), operative BMI (mean, 44 vs 43), and operative Excess Body Weight (EBW, kg, mean, 50 vs 50, *p* = 0.40). The EG had a higher Frailty Index according to the Memorial Sloan Kettering Frailty Index (mean, 1.68 vs 0.77, *p* < 0.01).

### Elderly Group vs Younger Group—Pre-Operative Obesity-Related Co-Morbidities

The elderly patients suffered from significantly higher pre-operative obesity-related co-morbidities in terms of diabetes mellitus (%, 49 vs 24, *p* < 0.01), hypertension (%, 73 vs 25, *p* < 0.01), dyslipidemia (%, 66 vs 33, *p* < 0.01), and obstructive sleep apnea (%, 21 vs 10, *p* < 0.01).

### Elderly Group vs Younger Group – Perioperative course – Table 2

**Table 2:** Elderly vs Younger groups: Perioperative and postoperative course, and long-term outcomes

Variable	Elderly Group *n* = 85	Younger Group *n* = 810	*p*—value
Minor morbidity (30-day Clavien-Dindo 1\2), n (%)	13 (15)	53 (6.5)	0.23
Major morbidity (30-day Clavien-Dindo 3\4), n (%)	3 (3.3)	27 (3.5)	0.75
90-day mortality, n (%)	0	0	
Post-operative leak, n (%)	1 (1.2)	16 (2)	0.60
% EBWL 6 months, mean (SD)	−60 (15)	−64 (18)	0.04
% EBWL 1 year, mean (SD)	−74 (18)	−82 (21)	< 0.01
% EBWL 2 years, mean (SD)	−72 (20)	−81 (23)	< 0.01
% EBWL 3 years, mean (SD	−64 (16)	−76 (23)	0.02
% EBWL 5 years, mean (SD	−53 (27)	−70 (29)	0.02
Post-operative Type II Diabetes Mellitus status - No amelioration, n (%) - Improved, Medication still needed, n (%) Resolved, n (%)	0 (0) 11 (48) 12 (52)	2 (3) 20 (27) 52 (70)	0.14
Post-operative Obstructive Sleep Apnea status - No amelioration, n (%) Resolved, n (%)	2 (22) 7 (78)	1 (4) 23 (96)	0.25
Post-operative Hypertension status - No amelioration, n (%) - Improved, Medication still needed, n (%) - Resolved, n (%)	2 (7) 10 (32) 19 (61)	7 (9) 19 (24) 52 (67)	0.67
Post-operative Dyslipidemia status - No amelioration, n (%) - Improved, Medication still needed, n (%) Resolved, n (%)	3 (11) 15 (53) 10 (36)	5 (7) 22 (31) 44 (61)	0.11

*SD* – standard Deviation, % *EBWL* –the percentage of Excess Body Weight Loss

Both groups had similar rates of minor post-operative morbidity according to Clavien – Dindo classification [23] of I/II (%, 15 vs 6.5, *p* = 0.23), major postoperative morbidity; Clavien – Dindo classification of III\IV (%, 3.3 vs 3. 5, *p* = 0.75), and no 90-days mortality. There was no statistically significant difference in leakage rates (%, 1.2 vs 2, *p* = 0.60).

### Elderly Group vs Younger Group – Procedural Outcomes; Weight Loss

**Fig.1:**
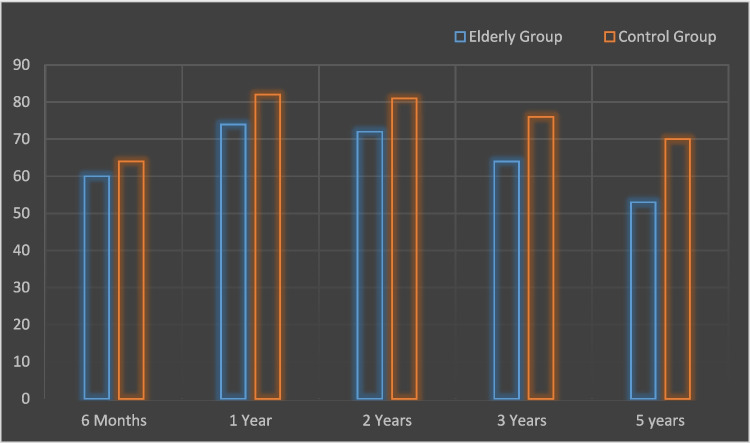
Comparison of changes of %EBWL following sleeve gastrectomy for 5 years follow-up

In terms of mean Percentage of Excess Body Weight Loss (%EBWL) in the six-month, one-year, two-year, three years, and five-year benchmarks, the results were significantly less favorable for the elderly group: 60 vs 64, 74 vs 82, 72 vs 81, 64 vs 76 and 53 vs 70 respectively. (Figure. 1).

### Elderly Group vs Younger Group – Procedural Outcomes; Obesity-Related Co-Morbidities Remission Rates (Figure. 2)

**Fig.2:**
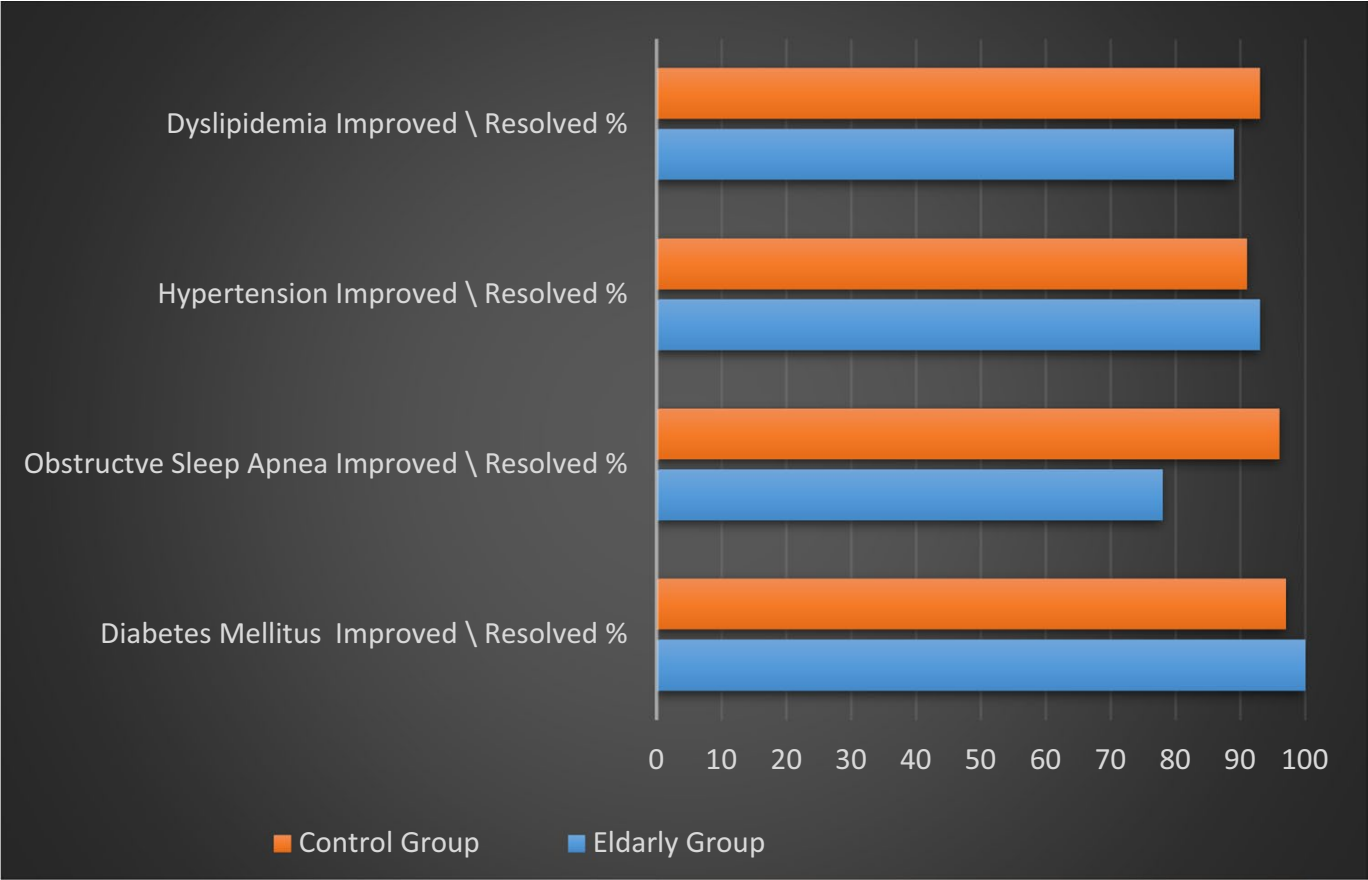
Comparison of changes in comorbidities following sleeve gastrectomy during 5 years follow-up

During long-term follow-up, there was a resolution or a notable improvement in co-morbidities in the vast majority of cases of both groups: Diabetes mellitus (%, 100 vs 97, *p* < 0.01), OSA (%, 78 vs 96, *p* = 0.05), Hypertension (%, 93 vs 91, *p* = 0.29) and dyslipidemia, (%, 89 vs 93, *p* = 0.11).

### Frail Group (FG) vs Non-Frail Group (NFG)—Demographics Table 3

**Table 3:** Frail vs non-Frail groups: Preoperative demographics and evaluation

Variable	Frail *n* = 60	Non-frail *n* = 835	*P* value
Gender, Female, n (%)	34 (57)	599 (72)	0.01
Age, mean (SD)	55 (8)	41 (13)	< 0.01
MSK-FI, mean (SD)	3.2 (0.4)	0.7 (0.8)	< 0.01
Weight at operation, KG, mean (SD)	119 (20)	119 (20)	0.80
BMI at operation, mean (SD)	44 (7)	43 (5)	0.62
EBW at operation, mean (SD)	51 (19)	41 (13)	0.80
Preoperative Diabetes Mellitus, n (%)	56 (93)	183 (22)	< 0.01
Preoperative Hypertension, n (%)	54 (90)	214 (26)	< 0.01
Preoperative Dyslipidemia, n (%)	48 (80)	276 (33)	< 0.01
Preoperative Obstructive Sleep Apnea, n (%)	18 (30)	85 (10)	< 0.01
HbA1c before operation, % mean (SD)	7.7 (1.4)	6.3 (1.3)	< 0.01

*SD* – Standard Deviation, *MSK-FI* – Memorial Sloan Kettering Frailty Index, *KG* – kilogram *BMI* – Body Mass Index, *EBW* – Excess Body Weight, HbA1c – Hemoglobin A1c

There were 60 patients in the FG compared to 835 in the NFG. Median age was 55(IQR, 51 – 60) for the FG vs 41(IQR, 31 – 52) for the NFG. Female gender prevalence was lower in the FG (%, 57 vs 72, *p* = 0.01). Pre-operative weight (kg, 119 vs 119, *p* = 0.80), BMI (mean, 44 vs 43, *p* = 0.62), and Excess Body Weight (kg, 51 vs 41, *p* = 0.80) were all comparable between the groups.

### Frail Group vs Non-Frail Group—Pre-Operative Obesity-Related Co-Morbdities

Patients in the FG suffered from higher rates of pre-operative obesity-related co-morbidities: diabetes mellitus (%, 93 vs 22, *p* < 0.01), hypertension (%, 90 VS 26,* p* < 0.01), OSA (%, 30 VS 10, *p* < 0.01), and dyslipidemia (%, 80 vs 33, *p* < 0.01).

### Frail vs Non-Frail Groups—Perioperative Course

The postoperative course was considerably more hazardous for frail patients; higher 30-day minor comorbidities (%, Clavien-Dindo of I/II, 13 vs 7, *p* < 0.01), higher 30-day major comorbidities (%, Clavien-Dindo of III/IV, 8 vs 3, *p* = 0.04), and almost double the post-operative leakage rates (%, 3 v 1.7, *p* = 0.09), however, it did not reach statistical significance. There were no 90-day mortality.

### Frail vs Non-Frail Groups—Procedural Outcomes; Weigh Loss: (Table 4)

**Table 4:** Frail vs non-frail groups: Perioperative and postoperative course, and long-term outcomes

Variable	Frail *n* = 60	Non-frail *n* = 835	*P* value
Minor morbidity (30-day Clavien-Dindo 1\2), n (%)	8 (13)	58 (7)	< 0.01
Major morbidity (30-day Clavien-Dindo 3\4), n (%)	5 (8)	25 (3)	0.04
90-day mortality, n (%)	0	0	
Post-operative leak, n (%)	3 (5)	1.7 (14)	0.09
%EBWL 6 months, mean (SD)	58 (20)	64 (17)	0.01
%EBWL 1 year, mean (SD)	73 (19)	82 (21)	< 0.01
%EBWL 2 years, mean (SD)	72 (21)	81 (22)	0.01
%EBWL 3 years, mean (SD)	63 (21)	76 (23)	0.20
%EBWL 5 years, mean (SD)	74 (24)	68 (29)	0.43
Post-operative Diabetes Mellitus status - Same, n (%) - Improved, n (%) - Resolved, n (%)	1 (3) 15 (52) 13 (45)	1 (2) 16 (23) 51 (75)	< 0.01
Post-operative Obstructive Sleep Apnea status - Same, n (%) - Resolved, n (%)	2 (22) 7 (78)	1 (4) 23 (96)	0.05
Post-operative Hypertension status - Same, n (%) - Improved, n (%) - Resolved, n (%)	2 (9) 9 (39) 12 (52)	7 (8) 20 (23) 59 (69)	0.29
Post-operative Dyslipidemia status - Same, n (%) - Improved, n (%) - Resolved, n (%)	3 (14) 13 (62) 5 (24)	5 (6) 24 (30) 50 (64)	< 0.01

*SD* – Standard Deviation, %*EBWL* –percentage of Excess Body Weight Loss

The mean Percentage of Excess Body Weight Loss was significantly lower for the frail group. In the six-month, year, and two-year benchmarks, the results were 58 vs 64, 73 vs 82, 72 vs 81, and 63 vs 76, respectively. However, in the third and fifth years post-operative, the percentage of EBWL proved to be similar: 63 vs 76 and 74 vs 68.

### Frail vs Non-Frail Groups—Procedural Outcomes; Obesity-Related Co-Morbidities Remission Rates

In our long-term follow-up, morbidity-related co-morbidities rates were significantly less favorable for patients in the FG when compared to the NFG. Diabetes mellitus resolution (%, 45 vs 75, *p* < 0.01) and improvement (%, 23 vs 52, *p* < 0.01) were lower for patients in the FG. The same results were observed with post-operative dyslipidemia resolution (%, 64 vs 24, *p* < 0.01) and improvement (%, 62 vs 30, *p* < 0.01) rates. OSA resolved in 56% of the FG vs 92% in the NFG (*p* = 0.05). Dyslipidemia resolved in 24% of frail patients vs 64% of the non-frail patients (*p* < 0.01). Hypertension status was comparable between the two groups.

## Discussion

Bariatric surgeries are effective in promoting sustained weight loss and controlling obesity-related comorbidities. This study aimed to validate the safety of LSG as a bariatric procedure for elderly and frail populations and assess its impact on long-term weight loss and remission of obesity-related comorbidities.

The use of bariatric surgeries for elderly populations has been primarily studied through meta-analyses and smaller cohort studies [20, 24–27]. Given that LSG is the most common bariatric and metabolic surgery procedure and the population is aging, verifying the long-term efficacy of LSG for elderly and frail patients is fundamental.

Over the past four decades, extensive research has demonstrated that bariatric surgeries are highly effective in improving obesity-related comorbidities. However, it is important to note that these positive effects may not always be permanent. For example, the SOS study [28] found that the remission rate for Type 2 Diabetes (T2D) after bariatric surgery was 72% at two years but decreased to 36% ten years later.

In our study of 85 elderly patients with T2D, we observed a full remission of 52% at an average follow-up of 29.5 months. Additionally, 48% of the patients experienced improved glycemic control while reducing the number and dosage of associated medications. These results are consistent with those seen in younger patient groups.

As previously reported [29–31], younger patients generally experience a greater percentage of excess body weight loss after bariatric surgery compared to older patients. Our findings align with this existing knowledge. Nevertheless, we observed that comorbidity remission was equally beneficial in the long term for both the young and elderly groups. Those findings are irrespective of the higher rates of weight regain in the elderly. We would like to suggest that weight loss may not be the sole factor for older patients undergoing bariatric surgery. The sustained improvement in comorbidities is equally important.

A comprehensive discussion with patients, carefully considering their motivations, priorities, and goals, can help ensure that they make informed decisions about bariatric surgery.

The Frailty index is a well-established predictor of perioperative complications [32]. A study published by Kolbe et al. a decade ago [33] has shown that frailty is an independent risk factor for serious morbidity and mortality in bariatric surgery patients. Another study published a few years later reinforced the impact of frailty on the safety of older patients undergoing bariatric surgery [34]. The Memorial Sloan Kettering Frailty Index has emerged as a valuable tool for assessing frailty. The MSK-fi incorporates various health domains, including functional status, nutritional deficiencies, and comorbid conditions, enabling a detailed assessment of the patient's operative risk. Studies have demonstrated that frailty, as measured by indexes such as the MSK-FI, is strongly associated with postoperative complications, prolonged recovery, and reduced long-term survival. MSK-FI was validated in different groups of patients who underwent surgical treatment, and its correlation to post-operative complications is well-established [3, 32, 35–37].

Frailty is a multidimensional syndrome that reflects a decline in physiological reserve across multiple organ systems, rendering patients more susceptible to adverse outcomes, including postoperative complications. The clinical relationship between frailty and these complications can be attributed to several interrelated mechanisms. As well described by Clegg et al. in 2013 [38], frail patients often exhibit chronic inflammation, impaired immune responses, and reduced anabolic capacity, which compromise their ability to heal and recover following surgical stress. Additionally, frailty is frequently associated with sarcopenia, diminished cardiovascular and pulmonary reserves, and increased prevalence of comorbidities, all of which exacerbate the risk of complications such as infections, delayed wound healing, and organ dysfunction [39].

These mechanisms collectively underline the elevated complication rates observed in frail patients and emphasize the importance of preoperative risk assessment and targeted interventions to mitigate these risks.

While the impact of frailty on perioperative safety in general, and specifically in the obese population, its impact on surgical efficacy has been less studied. One study published very recently [40] addresses specifically the correlation between frailty and postoperative weight loss. Their results at 1-year follow-up were favorable for the non-frail group.

Our study's data is in accordance with those findings. Firstly, we demonstrated significantly higher perioperative complications for frail patients, and additionally, our study discovered that efficacy is less favorable for frail patients. Our data adds significant information that has not been shown before, revealing that in addition to higher complication rates, frail patients also had lower success rates in terms of weight loss and obesity-related comorbidity resolution. This finding suggests that frailty may hold yet another critical factor determining the efficacy of bariatric surgery.

## Limitations

This study has several limitations inherent to its retrospective design, particularly due to the reliance on chart reviews that were not initially intended for research purposes. Consequently, some information may be missing or incomplete. To mitigate these biases, efforts were made to maintain the database prospectively. Nonetheless, a multi-center prospective trial is recommended to more comprehensively evaluate the impact of frailty on obese patients undergoing bariatric surgery.

Another notable limitation is the median follow-up period of three years, which may limit the strength and reliability of conclusions regarding long-term outcomes after bariatric surgery. Short follow-up durations often fail to capture the full range of postoperative effects, especially concerning weight maintenance and the resolution of comorbidities.

Additionally, the analysis did not incorporate multivariate adjustments for potential confounding factors that could influence the outcomes. This limitation should be taken into account when interpreting the results. Future studies with adjusted analyses are necessary to validate these findings.

## Conclusion

This study aimed to evaluate the safety and efficacy of Laparoscopic Sleeve Gastrectomy (LSG) in elderly and frail patients undergoing bariatric surgery. Our findings suggest that while LSG appears to be a generally safe and effective option for this population, frailty may influence the likelihood of perioperative complications and treatment outcomes. Furthermore, the relationship between weight loss and comorbidity resolution may be less straightforward in elderly patients, warranting careful consideration.

A thorough preoperative assessment, the implementation of prehabilitation protocols, and comprehensive postoperative care are recommended to help minimize perioperative risks and optimize outcomes.

## Data Availability

Data is provided within the manuscript or supplementary information files.
