# Correction: Frailty Index as a Predictor of Operative Safety and Efficacy in Patients Undergoing Laparoscopic Sleeve Gastrectomy

**DOI:** 10.1007/s11695-025-07819-3

**Published:** 2025-03-25

**Authors:** Eliahu Yonathan Bekhor, Boris Kirshtein, Noam Peleg, Nayyera Tibi, Hila Shmilovich, Lisa Cooper, Alex Tatarov, Nidal Issa

**Affiliations:** 1 Rabin Medical Center, Petah Tikva, Israel; 2 Tel Aviv University, Tel Aviv, Israel

**Correction: Obesity Surgery**

10.1007/s11695-025-07713-y

The published article has been revised to correct the spelling of author Nayyera Tibi.

